# Violation detection in power operation sites based on multi-scale detection and few-shot learning

**DOI:** 10.3389/frai.2026.1833234

**Published:** 2026-06-16

**Authors:** Yaokuan Wen, Jun Wang, Qiming Liu, Mo Zhou, Minzhe Tian

**Affiliations:** State Grid Henan Marketing Service Center, Zhengzhou, China

**Keywords:** few-shot learning, multi-scale detection, power operation, target detection, violation detection

## Abstract

**Introduction:**

Safety supervision at power operation sites is critical for ensuring worker safety and maintaining a reliable electricity supply. However, existing safety violation detection methods are constrained by limited labeled data, poor performance on small-object detection tasks, and interference from complex backgrounds.

**Methods:**

To overcome these challenges, this study proposes a framework that integrates multi-scale object detection with few-shot learning. A multi-scale feature extraction module is designed based on a feature pyramid network and channel attention mechanisms to enhance the perception of small objects. In addition, a few-shot learning framework incorporating a meta-learning strategy is introduced to address the scarcity of labeled safety violation samples and improve the model's adaptability to new tasks with limited training data.

**Results:**

Experimental results demonstrate that the proposed method consistently outperforms existing approaches across multiple evaluation metrics. The framework achieves notable improvements in small-object detection accuracy and few-shot learning performance, resulting in enhanced detection accuracy, robustness, and generalization capability.

**Discussion:**

The integration of multi-scale feature extraction and few-shot learning effectively addresses the challenges of safety violation detection in power operation environments. The proposed framework provides a practical and reliable solution for intelligent safety monitoring and has significant potential for real-world deployment in power operation sites.

## 1 Introduction

With the continuous enhancement of safety management in the power industry, ensuring a safe working environment has become a top priority. Various potential hazards are typically present in such environments, particularly when workers fail to wear the required protective equipment (such as safety helmets, gloves, etc.). These safety hazards pose a serious threat to the lives of frontline personnel. Once an accident occurs, it usually leads to serious consequences. Therefore, how to promptly and accurately identify safety violations at operation sites and implement effective prevention measures has become an important direction for improving safety management in power operations (Yan et al., 2022).

Traditional approaches based on manual monitoring and safety inspections suffer from significant limitations, including low efficiency, limited coverage, and susceptibility to human subjectivity. Especially in large-scale power operation sites, it is difficult for manual inspection to achieve real-time and comprehensive safety supervision. Consequently, the use of computer vision techniques for automated detection of safety violations has become an important direction for enhancing safety management in power operations (Wang et al., 2021; Zhang et al., 2024; Sanjeewani et al., 2024; Qian et al., 2025). In this paper, power operation sites refer to working environments involved in the construction, maintenance, inspection, and operation of electrical power systems. Workers are required to wear personal protective equipment (PPE), such as safety helmets and gloves, to ensure operational safety. Therefore, power operation safety violations in this study specifically refer to behaviors where required PPE is missing or improperly used during these activities. By analyzing on-site images, computer vision systems can detect in real time whether workers are wearing the required protective equipment, thereby issuing early warnings before accidents occur.

**Figure 1:**
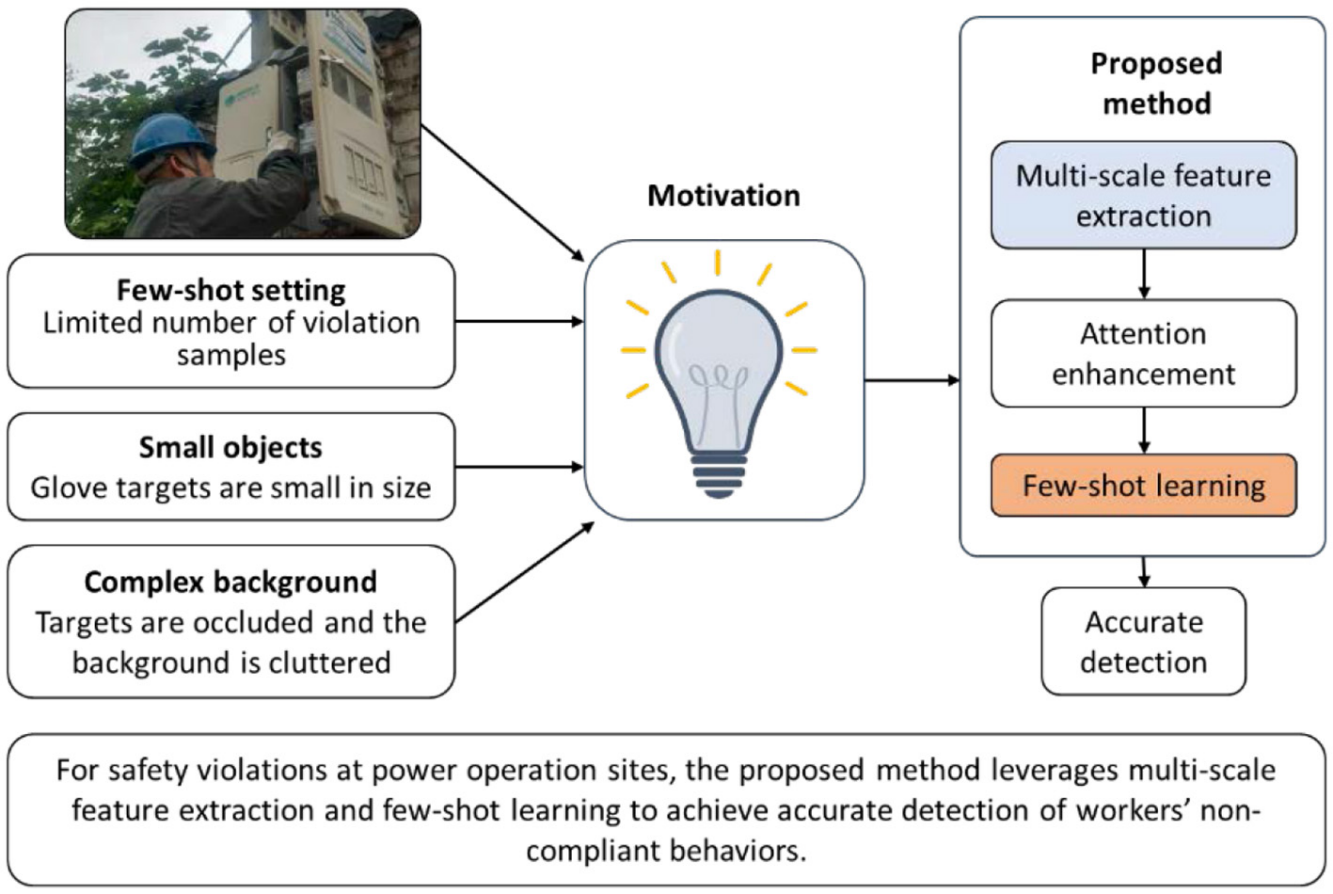
Motivation of this study.

However, safety violation detection at power operation sites faces multiple challenges. As illustrated in Figure 1, violations are typically rare, and instances in which workers are not wearing safety helmets or gloves occur infrequently, resulting in extremely scarce positive samples. Second, objects in power operation sites (such as gloves) are usually small in size, and may be partially occluded or embedded in complex backgrounds, which makes small-object detection a difficult task (Zhang et al., 2024). Conventional deep learning methods rely on large amounts of annotated data for training, but under few-shot conditions, they often fail to achieve satisfactory performance. In addition, the complex backgrounds in power operation images, along with variations in workers' poses and scene configurations, further increase the difficulty of detecting safety violations.

To tackle these problems, few-shot learning (FSL), a technique that enables models to learn from limited samples, has attracted considerable attention in recent years (Chen et al., 2024a,b; Gharoun et al., 2024). By learning how to rapidly adapt to new tasks with only a small amount of labeled data, few-shot learning can effectively alleviate the issue of data scarcity. In the context of power operation sites, violation samples are relatively rare, which makes few-shot learning an ideal solution. Moreover, to cope with the challenges arising from variations in object scale, multi-scale detection methods can extract features at different resolutions and enhance the detection capability for small objects (Zhu et al., 2024; Jiao et al., 2024; Rasheed and Zarkoosh, 2025). Multi-scale detection ensures that the model can effectively recognize and localize target objects regardless of their sizes.

In this paper, we propose a method that combines multi-scale object detection with few-shot learning for the automatic detection of safety violations at power operation sites. By extracting image features at multiple scales and integrating few-shot learning techniques, the model can accurately detect violations related to safety helmets, gloves, and other protective equipment, even with only a small amount of labeled data. Through the introduction of a multi-scale detection strategy, the proposed approach not only improves the detection accuracy of small objects, but also enhances robustness under complex backgrounds. The contributions of this work are as follows:

(1) A unified multi-scale few-shot detection framework for power operation scenarios. Unlike existing methods that address multi-scale detection and few-shot learning separately, this work integrates both into a unified framework, enabling effective detection of small and rare violation targets under limited annotation conditions.

(2) An attention-enhanced multi-scale feature extraction module. Built upon the Feature Pyramid Network (FPN), a channel attention mechanism is introduced to enhance target-relevant features. Compared with conventional multi-scale methods, this design improves robustness in complex backgrounds and significantly boosts small-object detection performance.

(3) An improved few-shot learning strategy with enhanced generalization. Different from standard few-shot detection approaches, this work adopts episodic meta-training combined with prototype augmentation, allowing the model to better capture intra-class variations and achieve more stable adaptation to scarce and imbalanced violation samples in real-world power operation environments.

The remainder of this paper is organized as follows. Section 2 reviews the literature related to this study. Section 3 presents the detailed design and implementation of the proposed method. Section 4 reports and analyzes the experimental results. Section 5 discusses the limitations of this work and outlines directions for future improvement.

## 2 Related work

### 2.1 Safety violation detection at power operation sites

In recent years, computer-vision-based safety monitoring techniques for work sites have attracted extensive attention. Early studies mainly relied on handcrafted features (such as HOG and SIFT) and traditional classifiers (such as SVM and Random Forest) to recognize workers and their personal protective equipment (PPE) (Huang et al., 2023; Zermane et al., 2023; Wang et al., 2018). However, due to their limited robustness in complex scenes, these methods struggle to adapt to the highly variable environments of power operation sites. With the development of deep learning, convolutional neural networks (CNNs) have demonstrated strong feature extraction capabilities in image detection tasks and have gradually been applied to detecting PPE such as safety helmets and gloves (Lee et al., 2023; Han and Zeng, 2021). For example, models based on Faster R-CNN or the YOLO family have achieved high-precision safety helmet detection in construction and industrial scenarios (Du et al., 2025; Vo et al., 2025; Yang et al., 2025). Nevertheless, such models generally rely on large amounts of annotated data for training and are not well-suited to class imbalance and few-shot scenarios. At power operation sites, violation samples are extremely scarce, and target objects often exhibit small size, which severely limit the effectiveness of existing methods in this domain.

### 2.2 Multi-scale object detection techniques

Multi-scale detection is one of the core approaches to handling significant variations in object size (Kim et al., 2019). Traditional single-scale detection methods often fail to balance the detection accuracy for both large and small objects when facing large scale differences. To address this issue, researchers have proposed multi-scale feature fusion architectures such as Feature Pyramid Networks (FPN), which integrate high-level semantic information with low-level detailed features through top-down feature propagation and lateral connections, thereby improving small-object detection performance (Chen et al., 2025). Subsequent structures such as PANet and BiFPN further enhance feature flow and cross-layer information interaction, enabling multi-scale detection models to perform more stably in complex scenes (Wu et al., 2023; Su et al., 2024). Multi-scale feature aggregation is used for UAV image object detection, which performs scale adaptation and information aggregation across multiple sets of feature maps, producing high-quality aggregated feature maps (Zhang et al., 2024, 2023). In safety monitoring scenarios, multi-scale detection can effectively improve the recognition of small-sized objects. However, when the amount of annotated data is limited, training multi-scale architectures may still be constrained by data scarcity, making it difficult for them to fully realize their potential.

### 2.3 Applications of few-shot learning in object detection

Few-Shot Learning (FSL) aims to address the problem under scarce annotations, with the core idea of enabling models to rapidly learn new categories from only a few samples (Song et al., 2023). Early work mainly focused on image classification tasks. Metric-learning-based methods such as Prototypical Networks and Relation Networks achieve few-shot classification by measuring the similarity between samples (Snell et al., 2017; Hu et al., 2018). Subsequently, meta-learning approaches such as MAML and Reptile were introduced into object detection, giving rise to the research direction of Few-Shot Object Detection (FSOD) (Xin et al., 2024; Yan et al., 2024; Du et al., 2025). These methods typically pretrain a detector on base categories and then fine-tune it with a small number of novel-category samples, thereby achieving rapid performance improvements under few-shot conditions. Existing studies have achieved remarkable progress on public datasets such as COCO and VOC. However, their application to real-world power operation sites remains challenging due to complex scenes, severe class imbalance, and a high proportion of small objects, which means that the generalization and adaptability of current models still need to be further improved.

### 2.4 Integrating multi-scale detection with few-shot learning

In recent years, researchers have begun exploring the integration of multi-scale detection with few-shot learning to jointly enhance feature representation capability and few-shot adaptability (Hussain et al., 2025). Some studies introduce meta-learning mechanisms into the detection head, enabling the model to learn class-agnostic discriminative features across multi-scale feature maps, thereby improving the detection performance for rare targets (Ye et al., 2025). Meta R-CNN, for instance, achieves fast transfer under few-shot conditions while maintaining competitive detection accuracy (Yan et al., 2019). In addition, other work incorporates attention mechanisms and feature enhancement modules to optimize feature selection and strengthen the model's perception of small objects (Xiong et al., 2024). Although these methods have achieved notable advances in general object detection, their application to power operation sites is still limited. Images in this domain are characterized by a high proportion of small objects, complex backgrounds, and extremely scarce violation samples. This calls for an adaptive detection framework that combines multi-scale feature extraction with few-shot learning to achieve high-precision and reliable recognition of unsafe behaviors.

In summary, existing research has made significant progress in object detection, few-shot learning, and multi-scale feature fusion. However, studies specifically targeting safety violation detection at power operation sites are still in their early stages. This paper proposes an integrated framework that combines multi-scale detection with few-shot learning, enabling high-accuracy and robust detection of small violation targets under limited annotation conditions, and providing a new solution for intelligent safety monitoring in power operation sites.

## 3 Methodology

To address the dual challenges of sample scarcity and small-object detection in safety violation detection at power operation sites, this paper proposes a detection framework that integrates multi-scale detection with few-shot learning. The proposed method introduces a multi-scale feature extraction mechanism to enhance the model's ability to perceive targets of different sizes, while leveraging few-shot learning strategies to enable the model to rapidly adapt to new detection tasks under limited annotation conditions. The overall framework is built upon a deep convolutional network and achieves high-precision detection of safety violations such as missing safety helmets and gloves through the joint optimization of a feature pyramid structure and a metric-learning module.

### 3.1 Overall framework

**Figure 2:**
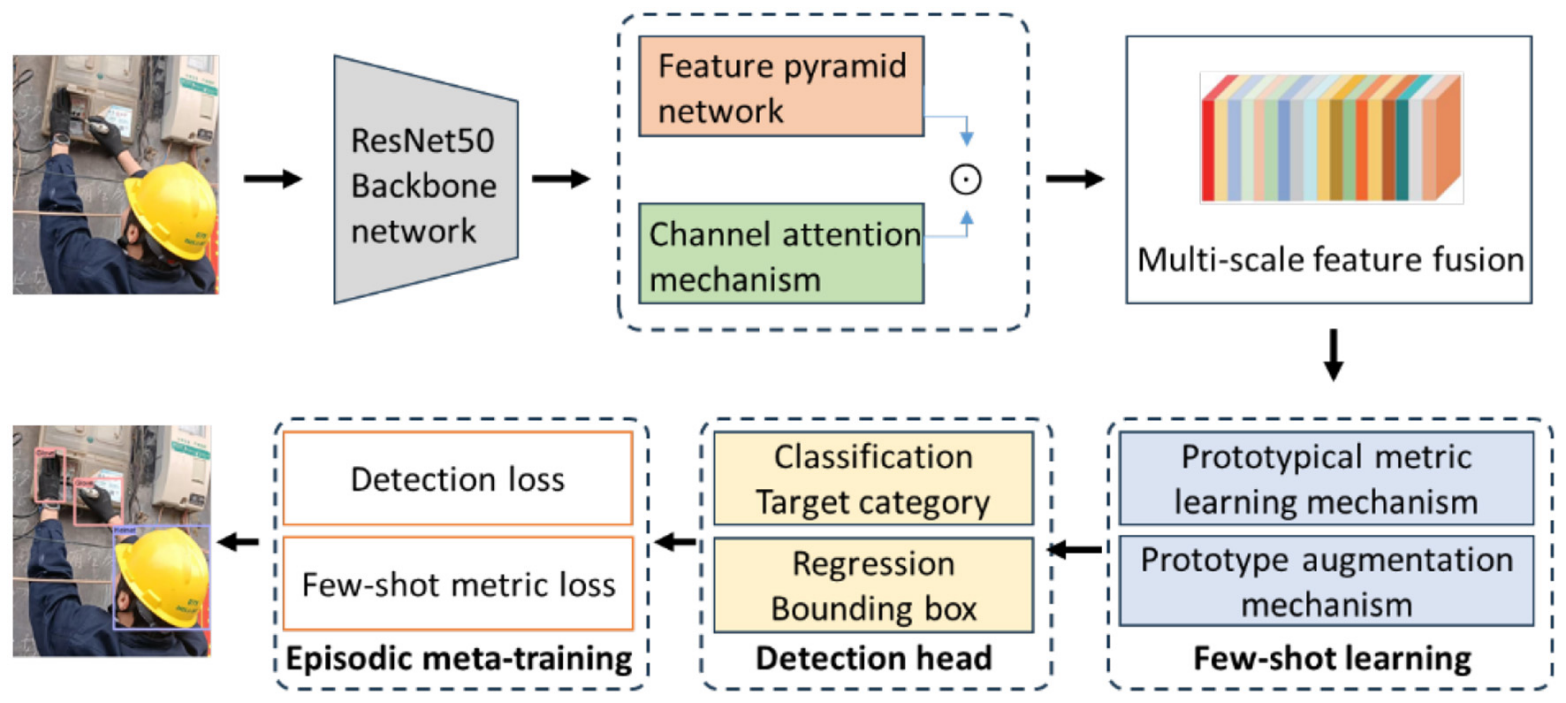
Overall architecture of the proposed method.

The overall framework proposed in this paper is shown in Figure 2 and consists of three core components: a multi-scale feature extraction module, a few-shot learning module, and the training and optimization strategy. The input image is first fed into the backbone network for feature extraction, producing feature maps at multiple scales. Subsequently, the multi-scale feature extraction module employs a Feature Pyramid Network (FPN) to fuse high-level semantic features with low-level spatial details, thereby generating feature representations enriched with multi-scale information. These fused features are then passed into the few-shot learning module, where a prototypical metric learning mechanism is introduced to compute similarities between samples and achieve category discrimination under few-sample conditions. Finally, the detection head outputs the target category and bounding box coordinates through classification and regression sub-networks.

During the training process, the backbone and feature fusion modules are jointly optimized, and an episodic training mechanism is incorporated to simulate few-shot tasks. This allows the model to be continually exposed to few-shot scenarios during training, thus enhancing its generalization ability in new environments. The framework effectively addresses the detection challenges in power operation sites, where few-shot samples, complex backgrounds, and multi-scale targets coexist.

### 3.2 Multi-scale feature extraction module

In power operation scenes, safety-related targets such as gloves and helmets often occupy only a small region of the image and are easily affected by occlusion, illumination changes, and background interference. Standard convolutional networks generate hierarchical feature maps, where shallow layers retain fine spatial details but contain weak semantic information, while deep layers contain stronger semantic features but lower spatial resolution. Therefore, directly using single-level features may lead to missed detection of small objects.

To address this issue, we construct a multi-scale feature extraction module based on a Feature Pyramid Network (FPN) and a channel attention mechanism. In this work, ResNet-50 is selected as the backbone network because it provides a good balance between detection accuracy and computational efficiency. Compared with lightweight networks, it offers stronger feature extraction for small and occluded objects, while requiring less data and computational cost than transformer-based backbones. In addition, its widespread use in object detection ensures stable performance and fair comparison with existing methods. Given an input image *X*, the backbone network ResNet-50 extracts feature maps from different stages:


$$\begin{array}{l} C_{i}=F_{i}\left(X\right),i\in \left\{2,3,4,5\right\}, \end{array}$$

where *C*_*i*_ denotes the feature map generated by the *i*-th stage of the backbone. The FPN fuses features in a top-down manner with lateral connections:


$$\begin{array}{l} P_{i}=Conv_{1\times 1}\left(C_{i}\right)+Up\left(P_{i+1}\right),i=2,3,4, \end{array}$$

where *Conv*_1 × 1_(·) is used for channel alignment and *Up*(·) denotes upsampling. A 3 × 3 convolution is then applied to each fused feature map to reduce aliasing effects:


$$\begin{array}{l} {\hat{P}}_{i}=Conv_{3\times 3}\left(P_{i}\right). \end{array}$$

Although FPN improves multi-scale representation, not all feature channels contribute equally to detecting safety violations. Some channels may respond strongly to background elements such as wires, tools, or equipment, while others are more relevant to helmets, gloves, and violation-related regions. Therefore, a channel attention mechanism is introduced to adaptively emphasize informative channels and suppress less useful ones. For each multi-scale feature map ${\hat{P}}_{i}\in {\mathbb{R}}^{C\times H\times W}$, global average pooling and global max pooling are first applied along the spatial dimensions:


$$\begin{array}{l} z_{avg}^{c}=\frac{1}{HW}\overset{H}{\underset{h=1}{\sum }}\overset{W}{\underset{w=1}{\sum }}{\hat{P}}_{i}^{c}\left(h,w\right), \end{array}$$

$$\begin{array}{l} z_{\max}^{c}=\underset{1\leq h\leq H,1\leq w\leq W}{\max}{\hat{P}}_{i}^{c}\left(h,w\right), \end{array}$$

where *c* denotes the channel index. Average pooling captures the global distribution of each channel, while max pooling highlights the most discriminative local response. The two descriptors are passed through a shared multilayer perceptron (MLP) to generate the channel attention weights:


$$\begin{array}{l} M_{c}\left({\hat{P}}_{i}\right)=\sigma \left(MLP\left(z_{avg}\right)+MLP\left(z_{\max}\right)\right), \end{array}$$

where σ(·) is the sigmoid activation function. The shared MLP consists of two fully connected layers:


$$\begin{array}{l} MLP\left(z\right)=W_{1}\delta \left(W_{0}z\right), \end{array}$$

where $W_{0}\in {\mathbb{R}}^{\frac{C}{r}\times C}$, $W_{1}\in {\mathbb{R}}^{C\times \frac{C}{r}}$, and δ(·) denotes the ReLU activation function. The reduction ratio reduces computational cost while preserving non-linear channel dependency modeling.

The final attention-enhanced feature map is obtained by channel-wise multiplication:


$$\begin{array}{l} {\tilde{P}}_{i}=M_{c}\left({\hat{P}}_{i}\right)⊗{\hat{P}}_{i}, \end{array}$$

where ⊗ denotes element-wise multiplication with broadcasting along the spatial dimensions. This operation assigns larger weights to channels that are more relevant to target detection and smaller weights to channels dominated by background noise.

The mathematical rationale of this mechanism is that global pooling transforms each feature map into compact channel descriptors, allowing the network to estimate the relative importance of different channels. The sigmoid function normalizes the attention weights into the range [0, 1], and the reweighting operation performs adaptive feature recalibration. As a result, the module enhances discriminative responses for small safety-related objects while suppressing irrelevant background features. The final multi-scale feature set is:


$$P=\left\{{\tilde{P}}_{2},{\tilde{P}}_{3},{\tilde{P}}_{4},{\tilde{P}}_{5}\right\},$$

which is used as the input to the subsequent few-shot learning module and detection head.

### 3.3 Few-shot learning module

In real-world power operation data, violation samples such as “NO-Helmet” and “NO-Glove” are extremely scarce. Under such conditions, conventional detection models are prone to overfitting and often fail to generalize. To address this issue, we introduce a few-shot learning mechanism that employs metric learning to enable rapid recognition of new categories from only a few samples.

During training, an episodic meta-learning strategy is adopted. In each episode, an *N*-way *K*-shot task is constructed, where *N* = 4 categories are sampled, each with *K*support samples (*K*∈{1, 5, 10}), along with *Q* = 10 query samples per category. All samples are processed through the shared backbone (ResNet-50) and the multi-scale feature extraction module to obtain embedding features. The embedding dimension is set to 256. After passing through the multi-scale feature extraction module, the feature representation of each sample is given by


$$\begin{array}{l} {\text{f}}_{i}^{k}=f_{feature}\left(x_{i}^{k}\right), \end{array}$$

where $x_{i}^{k}$denotes the *k*-th sample of class *i*, and *f*_*feature*_denotes the multi-scale feature mapping function.

For each category, the prototype is computed as the mean feature vector of its support samples:


$$\begin{array}{l} {\text{p}}_{i}=\frac{1}{K}\overset{K}{\underset{k=1}{\sum }}{\text{f}}_{i}^{k}. \end{array}$$

Given a query sample, classification is performed based on the Euclidean distance between its embedding and each class prototype. The probability distribution over classes is obtained via a Softmax function over the negative distances. For any query sample with feature **f**_*q*_, we compute its Euclidean distance to each class prototype:


$$\begin{array}{l} d_{i}\left({\text{f}}_{q}\right)=∥{\text{f}}_{q}-{\text{p}}_{i}{∥}_{2}^{2}. \end{array}$$

Based on the distance distribution, the probability that the sample belongs to class *i* is modeled as


$$\begin{array}{l} P\left(y=i|{\text{f}}_{q}\right)=\frac{\exp\left(-d_{i}\left({\text{f}}_{q}\right)\right)}{\overset{N}{\underset{j=1}{\sum }}\exp\left(-d_{j}\left({\text{f}}_{q}\right)\right)}. \end{array}$$

To improve robustness under intra-class variation, a prototype augmentation strategy is applied during training by adding Gaussian noise with a standard deviation of 0.01 to the class prototypes. Through this metric-learning-based classification and prototype augmentation, the model can not only effectively recognize existing categories under few-sample conditions but also rapidly adapt to new types of violations in power operation sites, thereby improving detection flexibility and robustness.

### 3.4 Training and optimization strategy

This paper adopts a joint training mechanism, in which object detection and few-shot metric learning are optimized within a unified end-to-end framework. The overall loss consists of a detection loss and a metric loss.

#### 3.4.1 Detection loss

The detection task simultaneously predicts the class distribution and the bounding-box location. Suppose a mini-batch contains *M*positive and negative samples, and the number of classes is *C*. The classification loss is defined as the multi-class cross-entropy:


$$\begin{array}{l} L_{cls}=-\frac{1}{M}\overset{M}{\underset{m=1}{\sum }}\overset{C}{\underset{c=1}{\sum }}y_{m,c}\log{ŷ}_{m,c} \end{array}$$

where *y*_*m, c*_is the one-hot label, and ŷ_*m, c*_is the predicted class probability. For bounding-box regression, we adopt the CIoU loss to enhance localization robustness:


$$\begin{array}{l} L_{box}=\frac{1}{M_{+}}\underset{m\in P}{\sum }\left(1-CIoU\left(b_{m},{\hat{b}}_{m}\right)\right), \end{array}$$

where Pdenotes the set of positive samples, and $M_{+}=|P|$. The overall detection loss is then given by


$$\begin{array}{l} L_{\det}=L_{cls}+\lambda_{1}L_{box}, \end{array}$$

where λ_1_is a weighting coefficient.

#### 3.4.2 Few-shot metric loss (prototype loss)

To encourage query samples to obtain high confidence for the correct class in the prototype-based metric space, we adopt the negative log-likelihood loss:


$$\begin{array}{l} L_{proto}=-\underset{q}{\sum }\log P\left(y_{q}|{\text{f}}_{q}\right), \end{array}$$

where *P*(*y*_*q*_∣**f**_*q*_)is given by the Softmax over negative distances as defined in the previous subsection. This loss shares the same underlying features with the detection head, ensuring consistency between the metric space and the detection feature space.

#### 3.4.3 Total loss and training strategy

The total loss function is defined as


$$\begin{array}{l} L_{total}=L_{\det}+\lambda_{2}L_{proto}, \end{array}$$

where λ_2_is a trade-off hyperparameter. Training is performed using episodic meta-training: in each iteration, an *N*-way *K*-shot support set and several query samples are sampled from the training classes, and are jointly optimized together with a standard detection mini-batch via backpropagation. Specifically, in our implementation, *N* = 4 is adopted to match the number of safety-related categories, while *K*ε{1,5,10} is varied to evaluate different few-shot conditions. The query set size is fixed to *Q* = 10 per class to ensure stable gradient estimation. During each episode, both support and query samples are passed through the shared backbone and multi-scale feature extraction module to obtain embedding representations. The AdamW optimizer is adopted, with an initial learning rate of 1 × 10^−4^and a weight decay of 1 × 10^−5^. The hyperparameters λ_1_and λ_2_are tuned on the validation set. This strategy ensures that the detection branch and the metric branch share a consistent set of discriminative features, and achieves stable performance gains under conditions where few-shot samples and small targets coexist.

## 4 Experiments and results

### 4.1 Experimental settings

#### 4.1.1 Experimental platform and implementation environment

All experiments are conducted on a high-performance GPU platform equipped with an NVIDIA RTX 4090 GPU. The software environment consists of Ubuntu 22.04 (Canonical Ltd., London, United Kingdom), Python 3.10 (Python Software Foundation Beaverton, Oregon, United States), PyTorch 2.2 (PyTorch Foundation, San Francisco, California, United States), and CUDA 12.1 (NVIDIA Corporation, Santa Clara, California, United States). The optimizer is AdamW, with an initial learning rate of 1 × 10^−4^, weight decay of 1 × 10^−5^, and momentum parameters β_1_ = 0.9, β_2_ = 0.999. Each experimental configuration is independently run three times, and the average performance is reported to reduce the impact of randomness. The hyperparameter settings are selected based on a combination of empirical experience and validation experiments. Specifically, the learning rate and weight decay follow commonly used configurations for AdamW to ensure stable convergence. Key parameters, including the loss weighting coefficients and few-shot settings (e.g., *K*-shot), are tuned on the validation set to achieve optimal performance. This strategy ensures a balance between training stability, generalization ability, and computational efficiency.

#### 4.1.2 Dataset and annotation setup

The experiments are conducted on a real-world image dataset collected from power operation sites, consisting of 5,000 high-resolution images covering diverse lighting, weather, and background conditions. All images are manually annotated by professional safety personnel, with labels stored in JSON format. The categories include “Helmet,” “NO-Helmet,” “Glove,” and “NO-Glove.” Among them, normal PPE-wearing samples account for approximately 90%, while violation samples (e.g., “NO-Helmet,” “NO-Glove”) account for only about 10%, reflecting the inherent rarity of unsafe behaviors in actual power operation scenarios. The dataset is split as follows:

- Training set: 3,200 images used for model training (including a small number of violation samples);
- Validation set: 800 images;
- Test set: 1,000 images covering diverse environments and lighting conditions.

To evaluate the adaptability of the few-shot learning module, only about 20% of the violation-class samples in the training set are retained for constructing few-shot tasks.

#### 4.1.3 Baselines

To validate the effectiveness of the proposed method, several representative detection models are selected as baselines for comparison on the power operation violation detection task. It should be noted that YOLOv5 is selected as the representative one-stage detector baseline, despite the availability of newer versions. This choice is motivated by the following considerations. First, YOLOv5 is a widely adopted and well-established benchmark in object detection research, with stable performance and extensive validation across various tasks, making it suitable for fair comparison. Second, many few-shot object detection frameworks and prior studies still use YOLOv5 or comparable architectures as baselines, ensuring consistency with existing literature.

- Faster R-CNN: A classical two-stage object detection baseline.
- YOLOv5: A one-stage multi-scale detection model.
- RetinaNet: A detector that incorporates a feature pyramid structure and Focal Loss.
- FSOD-TFA: A typical few-shot object detection model with strong rapid adaptation capability.
- Meta R-CNN: A few-shot detection framework that integrates meta-learning strategies.

#### 4.1.4 Evaluation metrics

To comprehensively assess model performance, the following quantitative metrics are adopted:

- Mean Average Precision (mAP@0.5): Average detection precision at an IoU threshold of 0.5. For each category, Average Precision (AP) is computed as the area under the Precision–Recall curve:

$$\begin{array}{l} AP=\overset{1}{\underset{0}{\int }}P\left(R\right)dR, \end{array}$$

where *P*(*R*)denotes Precision as a function of Recall. The mean Average Precision (mAP) is obtained by averaging AP over all categories:


$$\begin{array}{l} mAP=\frac{1}{C}\overset{C}{\underset{c=1}{\sum }}AP_{c}. \end{array}$$

- Small-object mAP (mAPs): Average precision computed for targets with an area smaller than 32^2^ pixels, used to evaluate small-object detection capability.
- Precision and Recall: Measuring detection accuracy and completeness, respectively. Precision and Recall are defined as:

$$\begin{array}{l} Precision=\frac{TP}{TP+FP},\;Recall=\frac{TP}{TP+FN}, \end{array}$$

where *TP*, *FP*, and *FN*denote the number of true positives, false positives, and false negatives, respectively.

- F1-score: A holistic performance indicator that reflects the balance between Precision and Recall.

$$\begin{array}{l} F1=2\cdot \frac{Precision\cdot Recall}{recision+Recall}. \end{array}$$

#### 4.1.5 Training details

During training, the batch size is set to 8, and the total number of epochs is 50. In each training episode, categories and samples are randomly sampled to ensure sufficient coverage of different classes and scenes. After training, the model parameters corresponding to the best performance on the validation set are selected for final testing. For optimization, the AdamW optimizer is adopted with an initial learning rate of 1 × 10^−4^and a weight decay of 1 × 10^−4^. The learning rate is gradually decreased using a cosine annealing schedule. In the few-shot learning module, episodic training is performed using an *N*-way *K*-shot setting, where *N* = 4, *K*∈{1, 5, 10}, and the number of query samples per class is set to 10. To stabilize training and prevent overfitting under limited samples, DropBlock regularization is applied to the multi-scale feature layers with a drop probability of 0.1. Gradient clipping with a maximum norm of 5 is used to avoid gradient explosion. Early stopping is employed based on validation mAP, with a patience of 5 epochs.

### 4.2 Experimental results and analysis

Experiments on the power operation site image dataset are conducted to systematically validate the proposed multi-scale few-shot detection framework. The experiments include overall performance comparison, analysis of small-object detection capability, evaluation of few-shot learning performance, and ablation studies.

#### 4.2.1 Overall detection performance comparison

Table 1 presents the performance comparison between the proposed method and several mainstream detection models on the test set. It can be observed that the proposed approach achieves higher overall accuracy than traditional detection networks, while maintaining stable performance under few-shot conditions.

**Table 1:** Detection performance comparison of different methods on the test set.

Methods	mAP@0.5 (%)	mAPs (%)	Precision (%)	Recall (%)	F1-score (%)
Faster R-CNN	81.2	64.8	84.5	78.3	81.3
YOLOv5	83.7	66.1	86.9	79.4	83.0
RetinaNet	84.5	67.8	87.2	80.5	83.7
Meta R-CNN	85.2	69.3	88.0	81.2	84.5
FSOD-TFA	86.0	70.8	89.3	82.0	85.5
**Proposed**	**87.8**	**74.2**	**90.4**	**83.5**	**86.8**

The bold values highlight the performance of the proposed method.

From Table 1, it can be observed that the proposed method achieves the best performance across evaluation metrics. This improvement can be attributed to the joint optimization of multi-scale feature extraction and few-shot learning.

Compared with traditional detectors such as Faster R-CNN and RetinaNet, the proposed method demonstrates superior accuracy, particularly in mAPs, indicating its enhanced capability in detecting small objects. This is mainly due to the multi-scale feature extraction module, which effectively fuses semantic and spatial information across different resolutions and highlights target-relevant features through the channel attention mechanism.

Compared with few-shot detection methods such as Meta R-CNN and FSOD-TFA, the proposed method achieves further performance gains. This is because the introduced episodic meta-training and prototype augmentation enable the model to better capture intra-class variations and improve generalization under limited violation samples. In contrast, existing few-shot methods often rely on standard feature representations and lack specialized mechanisms for handling small objects in complex scenes.

In addition, the proposed method achieves a better balance between precision and recall, as reflected by the highest F1-score. This indicates that the model not only improves detection accuracy but also reduces missed detections, which is critical for safety monitoring in power operation sites. Overall, the results validate the effectiveness of integrating multi-scale detection and few-shot learning for handling small, sparse, and complex targets in real-world scenarios.

#### 4.2.2 Efficiency comparison

We evaluate the computational efficiency and model complexity of different methods. Specifically, inference speed is measured in frames per second (FPS) on an NVIDIA RTX 4090 GPU under a unified input resolution, and the number of model parameters (Params) is used to reflect model complexity. The comparison results are summarized in Table 2.

**Table 2:** Computational efficiency and model complexity of different methods.

Methods	FPS	Params (M)
Faster R-CNN	38.6	41.3
YOLOv5	112.4	7.2
RetinaNet	52.8	36.5
Meta R-CNN	31.7	45.8
FSOD-TFA	34.2	43.6
**Proposed**	**46.5**	**39.8**

The bold values highlight the performance of the proposed method.

Table 2 compares the inference speed and parameter complexity of different methods. YOLOv5 achieves the highest FPS due to its lightweight one-stage detection structure, while two-stage few-shot methods such as Meta R-CNN and FSOD-TFA show lower inference speed because of their additional meta-learning branches. The proposed method reaches 46.5 FPS with 39.8M parameters, indicating that the introduced multi-scale feature extraction and few-shot learning modules improve detection accuracy while maintaining acceptable computational efficiency for practical deployment.

#### 4.2.3 Analysis of small-object detection capability

To evaluate the impact of the multi-scale feature extraction module on small-object detection, we separately compute the detection results for targets of different scales. In this study, object scales are defined based on the pixel area of the ground-truth bounding boxes. Specifically, following common practice in object detection, objects are categorized as small, medium, and large according to their bounding-box area: small objects correspond to targets with an area smaller than 32^2^ pixels, medium objects fall within the range of 32^2^ to 96^2^ pixels, and large objects exceed 96^2^ pixels. In the context of power operation scenes, gloves and partially visible helmets are predominantly categorized as small objects due to their limited spatial extent, while fully visible helmets and human upper-body regions typically fall into the medium-object category. Large objects mainly correspond to prominent human instances or close-range subjects occupying a substantial portion of the image. The corresponding results are summarized in Table 3.

**Table 3:** mAP comparison across different object sizes.

Methods	Small objects (mAPs)	Medium objects (mAPm)	Large objects (mAPl)
YOLOv5	66.1	84.5	88.3
RetinaNet	67.8	85.1	89.0
Meta R-CNN	69.3	86.0	89.2
**Proposed (ours)**	**74.2**	**86.8**	**89.4**

The bold values highlight the performance of the proposed method.

The results show that the proposed method achieves significantly better performance than other approaches in small-object detection. In particular, for small targets, the combination of multi-scale feature extraction and the channel attention mechanism effectively enhances the model's ability to recognize small objects. The underlying mechanism is that channel attention adaptively reweights feature channels based on global contextual information, thereby emphasizing channels that are more relevant to small targets while reducing the influence of background-dominated channels. Multi-scale feature extraction enables the network to capture key information about targets at different resolutions, thereby improving its perception of small objects, while the channel attention mechanism further strengthens the selective focus on target-related features, reducing the impact of background noise on small-object detection.

It is worth noting that the proposed method performs on par with other approaches for medium and large objects, indicating that the multi-scale detection framework does not degrade the detection performance of larger targets. Instead, by improving the detection of small objects, it enhances the overall performance of the model across different scenarios.

#### 4.2.4 Analysis of few-shot learning capability

We further investigate the model's performance under different numbers of training samples. To this end, experiments are conducted under 1-shot, 5-shot, and 10-shot settings, and the results are summarized in Table 4.

**Table 4:** Detection performance under different numbers of samples.

Number of samples (K-shot)	mAP@0.5 (%)	Precision (%)	Recall (%)
1-shot	82.1	84.0	77.6
5-shot	86.2	88.5	82.1
10-shot	**87.8**	**90.4**	**83.5**

The bold values highlight the performance of the proposed method.

From Table 4, it can be observed that the model performance improves progressively as the number of samples increases. Under the 1-shot setting, although the number of support samples is extremely limited, the model is still able to maintain reasonably good performance, indicating that the few-shot learning module can rapidly learn and perform effective classification from very scarce data. Under the 5-shot and 10-shot settings, the model performance is further enhanced.

#### 4.2.5 Ablation study

**Figure 3:**
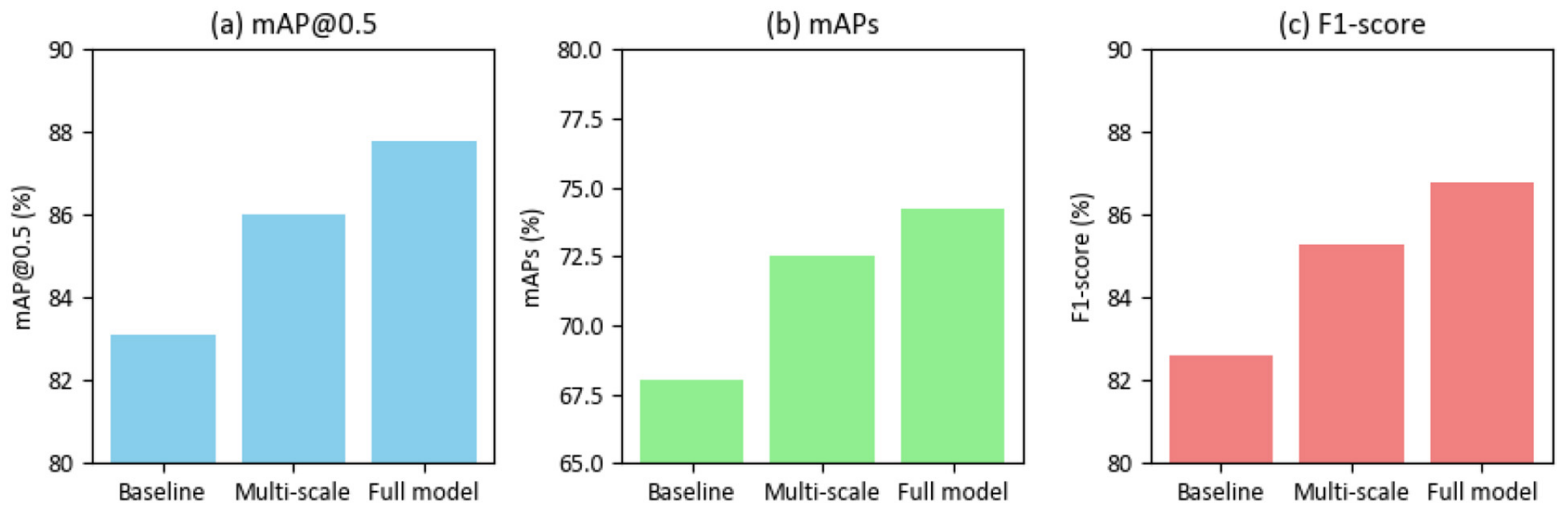
Figure 3. Ablation study results: **(a)** mAP@0.5 (%), **(b)** mAPs (%), and **(c)** F1-score (%) for the Baseline, Multi-scale, and Full model configurations.

To verify the contribution of each module to the overall performance, we conduct an ablation study by comparing model variants with different components removed. The corresponding results are reported in Table 5 and illustrated in Figure 3.

**Table 5:** Ablation study results of different modules.

Model architecture	mAP@0.5 (%)	mAPs (%)	F1-score (%)
Baseline (without multi-scale module, without FSL)	83.1	68.0	82.6
+ Multi-scale feature extraction module	86.0	72.5	85.3
+ Few-shot learning module (full model)	**87.8**	**74.2**	**86.8**

The bold values highlight the performance of the proposed method.

From Table 5, it can be seen that removing the multi-scale feature extraction module leads to a clear drop in small-object detection performance (mAPs), indicating that multi-scale feature extraction plays a crucial role in detecting small targets. After introducing the few-shot learning module, the overall performance is further improved, which confirms the effectiveness of the few-shot learning module under data-scarce conditions. These two modules complement each other and jointly enhance the detection accuracy and robustness of the model.

## 5 Discussion and limitations

Although the proposed method achieves strong performance in safety violation detection at power operation sites, several limitations remain. First, the dataset size is relatively limited, especially for violation samples, which may affect the model's generalization ability in unseen scenarios. Second, the framework is mainly designed for power operation environments, and its performance in other industrial domains may require additional fine-tuning or domain adaptation. In addition, the current method relies on supervised annotations and may still experience performance degradation under severe occlusion or low-light conditions.

Future work will focus on expanding the dataset scale, improving cross-domain adaptability, and exploring self-supervised and lightweight real-time detection methods to further enhance practical deployment performance.

## 6 Conclusion

This paper proposes an innovative method that integrates multi-scale object detection with few-shot learning to address key challenges in safety violation detection at power operation sites. Specifically, a multi-scale feature extraction module is introduced to effectively enhance the detection capability for small objects, demonstrating clear advantages in complex backgrounds, partially occluded scenarios, and small-object detection tasks. At the same time, through a few-shot learning framework, the model maintains good generalization ability under scarce annotation conditions and can rapidly adapt to new classes of detection tasks. Experimental results show that the proposed framework outperforms existing baseline methods across multiple evaluation metrics.

Despite its strong practicality and satisfactory performance in detecting safety violations at power operation sites, the proposed approach still leaves room for improvement. Future work may explore more efficient training strategies, such as self-supervised learning and transfer learning, to further reduce reliance on labeled data and to optimize real-time detection performance under extreme conditions. In addition, considering the highly dynamic and variable environments in power operation scenarios, further enhancing the model's robustness across different environmental conditions will be an important direction for future research.

In summary, this work provides an efficient and reliable solution for intelligent safety monitoring in power operation sites and holds substantial practical application value. By appropriately combining multi-scale detection with few-shot learning, the proposed method offers an effective perspective for both industry and academia, and promotes the evolution of safety management in the power sector toward greater intelligence and automation.

## Data Availability

The raw data supporting the conclusions of this article will be made available by the authors, without undue reservation.
